# Supplementary material for: Genome-Wide Identification, Expression Analysis, and Subcellular Localization of Carthamus tinctorius bHLH Transcription Factors
Source: Int J Mol Sci. 2019 Jun 21;20(12):3044. doi: 10.3390/ijms20123044 (PMC6627405; doi:10.3390/ijms20123044)
Supplement: Supplementary file 1 [file ijms-20-03044-s001.pdf]

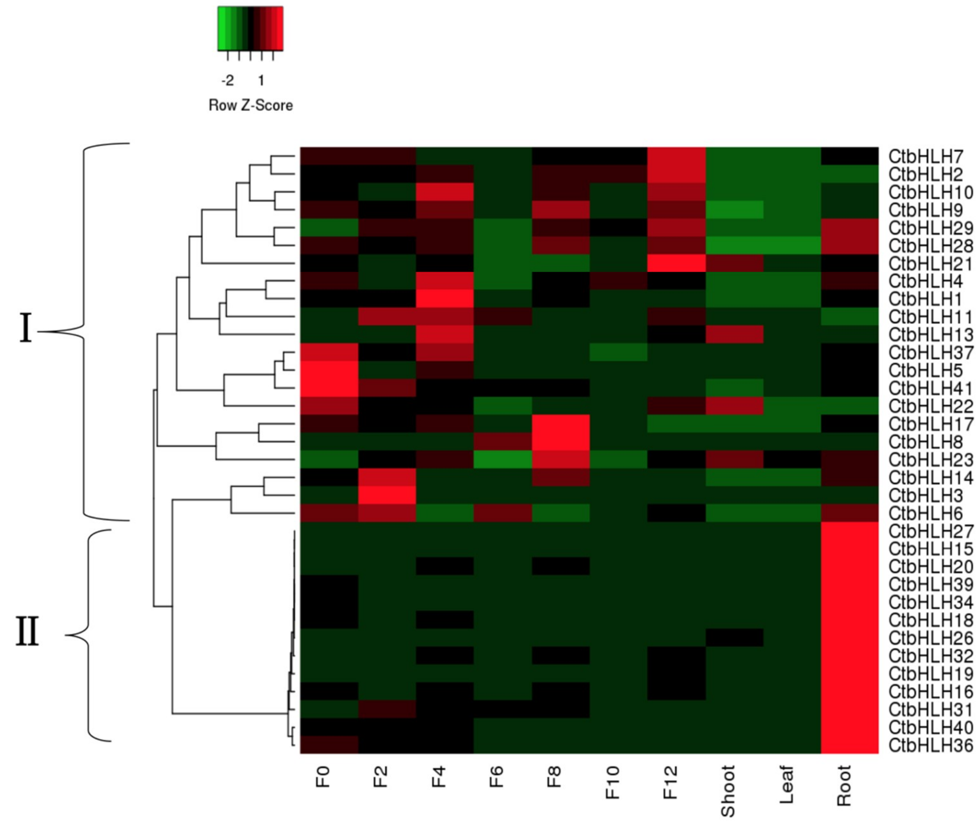

Figure S1. Schematic pattern of tissue-specific gene expression of Carthamus tinctorius Bhlh members. Four tissues, including roots, shoots, leaves, and 0-, 2-, 4-, 6-, 8-, 10-, and 12-day old petals were determined. The scale bars represent the log2 transformations of the RPKM values.
